# Clostridial Diseases of Horses: A Review

**DOI:** 10.3390/vaccines10020318

**Published:** 2022-02-17

**Authors:** Francisco A. Uzal, Mauricio A. Navarro, Javier Asin, Eileen E. Henderson

**Affiliations:** 1 California Animal Health and Food Safety Laboratory, San Bernardino Lab, University of California-Davis, San Bwernardino, CA 92408, USA; jasinros@ucdavis.edu (J.A.); eehenderson@ucdavis.edu (E.E.H.); 2 Instituto de Patología Animal, Facultad de Ciencias Veterinarias, Universidad Austral de Chile, Valdivia 5110566, Chile; mauricio.navarro@uach.cl

**Keywords:** botulism, clostridial diseases, colitis, enteritis, enterocolitis, gas gangrene, horses, infectious necrotic hepatitis, tetanus, Tyzzer disease, review

## Abstract

The clostridial diseases of horses can be divided into three major groups: enteric/enterotoxic, histotoxic, and neurotoxic. The main enteric/enterotoxic diseases include those produced by *Clostridium perfringens* type C and *Clostridioides difficile*, both of which are characterized by enterocolitis. The main histotoxic diseases are gas gangrene, Tyzzer disease, and infectious necrotic hepatitis. Gas gangrene is produced by one or more of the following microorganisms: *C. perfringens* type A, *Clostridium septicum, Paeniclostridium sordellii*, and *Clostridium novyi* type A, and it is characterized by necrotizing cellulitis and/or myositis. Tyzzer disease is produced by *Clostridium piliforme* and is mainly characterized by multifocal necrotizing hepatitis. Infectious necrotic hepatitis is produced by *Clostridium novyi* type B and is characterized by focal necrotizing hepatitis. The main neurotoxic clostridial diseases are tetanus and botulism, which are produced by *Clostridium tetani* and *Clostridium botulinum*, respectively. Tetanus is characterized by spastic paralysis and botulism by flaccid paralysis. Neither disease present with specific gross or microscopic lesions. The pathogenesis of clostridial diseases involves the production of toxins. Confirming a diagnosis of some of the clostridial diseases of horses is sometimes difficult, mainly because some agents can be present in tissues of normal animals. This paper reviews the main clostridial diseases of horses.

## 1. Introduction

Members of the genus *Clostridium* are anaerobic, mostly Gram-positive sporulated rods. Although the majority of the members of the genus are non-pathogenic commensal or soil bacteria, several clostridial species are responsible for some of the major diseases of humans and animals, including enteric and enterotoxemic syndromes, blackleg, gas gangrene, clostridial hepatitis, tetanus and botulism [1,2].

Clostridial diseases can be divided into three major groups: enteric/enterotoxic, histotoxic and neurotoxic diseases. The pathogenesis of clostridial diseases involves the production of powerful toxins. However, most of them are infectious diseases, which means that the clostridia responsible for disease need to infect the host, overcome host immune defenses, grow, multiply in tissues and elaborate their toxins [1]. The exception to this mechanism is botulism, which is most frequently a true toxemia in which the host ingests preformed botulinum toxin in the food [2].

All the pathogenic clostridia have the ability to sporulate, which is the process to produce resistant spores. These structures are resistant to heat, desiccation, and other environmental factors, which allow them to survive environmental adversities, until conditions are more favorable to bacterial growth and multiplication. Sporulation is crucial in the epidemiology of most clostridial infections, as it allows the microorganism to bypass the need to be passed from one host animal to another in vegetative form [1,2].

Horses, like many other animal species, are affected by a variety of clostridial diseases, but most of them have specific epidemiological, clinical, and pathological characteristics, which are essential at the time to make a diagnosis and institute measures of control and treatment. We review here the main clostridial diseases of horses.

## 2. Enteric and Enterotoxic Clostridial Infections

Enteric clostridial diseases are those in which lesions occur in the intestine, while enterotoxic diseases are those in which the clostridial toxins are produced in the intestine but are then absorbed into the general circulation and exert their effects in distant organs such as the brain or lungs. While many clostridial diseases are both enteric and enterotoxic, a few of them are only enteric and others are only enterotoxic.

### 2.1. Diseases Produced by Clostridium perfringens

#### 2.1.1. General Characteristics of *Clostridium perfringens*

*Clostridium perfringens* is a Gram-positive, anaerobic and sporulated rod, which is found ubiquitously in the environment and the intestinal contents and feces of most humans and animals [3]. Diseases produced by this microorganism are mediated by a vast array of toxins, six of which (alpha (CPA), beta (CPB), epsilon (ETX), iota (ITX), enterotoxin (CPE) and necrotic enteritis like B toxin (NetB)) are used to classify *C. perfringens* into seven toxinotypes (Table 1; [4]).

While the epidemiology of the diseases produced by each of the toxinotypes of *C. perfringens* has its own peculiarities, which are discussed under each toxinotype below, there are some general characteristics that apply to all or most of those diseases. Amongst these is the fact that all toxinotypes of *C. perfringens* may be found, at variable prevalence, in the intestinal content of normal individuals of several animal species. Because of this, different predisposing factors, discussed below, are required for each disease to occur. In addition, all enteric diseases produced by *C. perfringens* are non-contagious infections for which the organisms need to multiply in the alimentary tract and produce toxins, which are the most significant virulence factors of these microorganisms. Finally, these diseases tend to occur sporadically or, at the most, in the form of small clusters, with a relatively low incidence.

#### 2.1.2. Diseases Produced by *Clostridium perfringens* Type B

*C. perfringens* type B isolates carry the genes encoding CPA, CPB and ETX toxins, although different strains can also encode several other toxins, which are not used in the typing of this microorganism. The two main virulence factors of type B strains are, however, CPB and ETX [5].

Infections by this microorganism have been described, mostly in ruminants, in the Middle East, Europe, South Africa and New Zealand [6,7]. To the best of our knowledge, no cases of this infection have been reported from the Americas. Type B disease seems to be very rare in foals [6,7,8], so only a brief reference to the disease will be presented here.

The disease is clinically characterized by acute abdominal pain, distended abdomen and hemorrhagic diarrhea in foals within the first few days of life. Intestinal gross and microscopic lesions associated with infection by *C. perfringens* type B are very similar to those produced by *C. perfringens* type C (see below) and are characterized by severe diffuse or multifocal to coalescing necrohemorrhagic or ulcerative enteritis [6,8].

A presumptive diagnosis of type B infection can be established by clinical signs and gross and microscopic changes. A final diagnosis, however, should be established based on the detection of both CPB and ETX in the intestinal content of affected animals. In places where *C. perfringens* type B has not been isolated before, isolation of this microorganism (especially in pure culture) from the intestinal content or feces of animals with compatible clinical signs and lesions should be considered a very good diagnostic indicator of type B infection [6,9].

#### 2.1.3. Diseases Produced by *Clostridium perfringens* Type C

**Etiology**. Strains of *C. perfringens* type C encode CPA and CPB toxins [10,11]. It has been determined that CPB, a necrotizing toxin that forms pores in affected cells, is the primary driver of the clinical signs and lesions observed in animals infected with *C. perfringens* type C [10,11]. This toxin is thermolabile and sensitive to proteases (particularly trypsin). Additionally, some strains of *C. perfringens* type C are also documented to produce other toxins including CPE, beta2 (CPB2), perfringolysin O (PFO), and large clostridial toxin (TpeL) [12].

**Epidemiology.***C. perfringens* type C associated disease (often termed necrotic enteritis) occurs most commonly in neonatal animals [12]. This is attributed in large part to the trypsin inhibitors in colostrum resulting in low levels of intestinal trypsin activity in the intestine of neonatal animals. In horses, *C. perfringens* type C associated disease is most common in foals, with sporadic cases reported in adults [10,13].

**Pathogenesis.***C. perfringens* type C may be found in soil and feces of humans and animals [12]. Spores can persist in the environment for extended periods of time. Infection can occur due to environmental contamination or exposure to infected feces. It has also been postulated that foals can become infected from the teats of their dams [13]. In foals, the presence of trypsin inhibitors in colostrum prevents the lysis of CPB; the pathogenesis of *C. perfringens* type C-associated disease in adult animals is less clear. Regardless of age, the intestinal mucosa is colonized by the bacterium and production and secretion of toxins (specifically CPB) follows. CPB induces cell death through the creation of pores in the host cell membrane resulting in an efflux of K+ and influx of Na^+^, Cl^−^, and Ca^2+^ [12]. CPB has been shown to bind to endothelial cells resulting in vascular damage, thrombosis, and necrosis. Death in affected animals is thought to be the result of systemic circulation of this toxin associated with the enteric lesions [10].

**Clinical signs**. The clinical course of the disease is typically peracute to acute, although subacute to chronic disease can occur occasionally. Affected horses can present with colic, lethargy, depression, pyrexia, and/or diarrhea (often bloody) [10,12]. In more chronically affected animals, clinical signs often include diarrhea, dehydration, weight loss, and weakness. Neurologic signs and sudden death can also occur [13].

**Figure 1:**
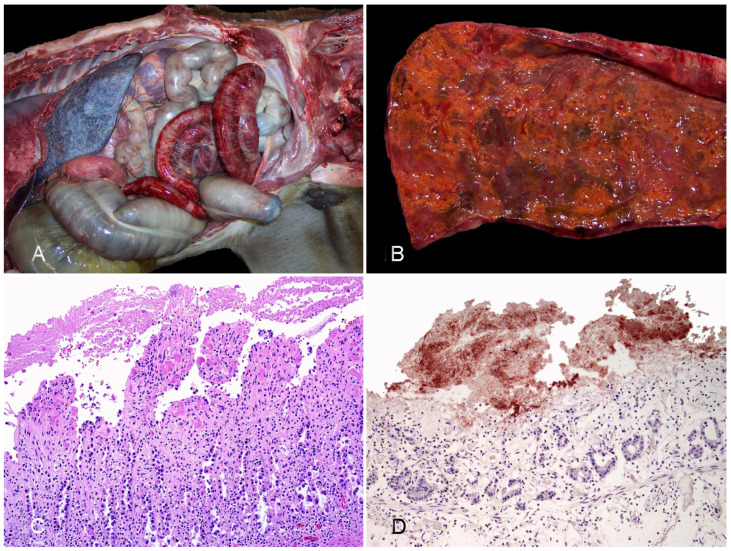
Neonatal foal with enterocolitis produced by *Clostridium perfringens* type C. (**A**) The serosal view of a large segment of the small intestine is diffusely congested and emorrhagic and the serosa of the large colon and cecum are dark. (**B**) The mucosa of the small intestine is necrotic and covered by an orange pseudomembrane. (**C**) The small intestine shows diffuse mucosal necrosis and multifocal thrombosis (arrowheads); a pseudomembrane (pm) is observed in the lumen; HE. (**D**) Strong positivity for *C. perfringens* is observed in the superficial mucosa and lumen of the small intestine; *C. perfringens* IHC. Reprinted with permission from ref. [10]. Copyright 2012 Vet Pathol.

**Gross pathology.** Gastrointestinal lesions can be segmental or diffuse and affect any portion of the intestinal tract (small intestine, cecum, or colon) [13]. Gross lesions include hyperemia/hemorrhage of the mucosa and serosa, transmural thickening, edema, emphysema, ulceration, and occasionally pseudomembrane formation (Figure 1A,B) [10]. In affected segments of the gastrointestinal tract, contents are often red to brown, watery, and fetid. Extraintestinal lesions are consistent with endotoxemia and septicemia and include petechiae or ecchymoses on the intestinal serosa, endocardium, pericaridum, pleura and mesentery; serous/serosanguineous pericardial, pleural, or peritoneal effusion; and pulmonary congestion and edema [12].

**Histopathology.** Microscopic lesions associated with *C. perfringens* type C infection are characterized by a hemorrhagic and necrotizing enteritis and/or colitis [12,13]. In affected sections of the intestine, there is coagulative necrosis of the intestinal mucosa with loss of normal villous architecture and the mucosal epithelium, collapse of the crypts, hemorrhage, congestion, edema, and mucosal or submucosal thrombosis (Figure 1C) [10,13]. Fibrinonecrotic pseudomembranes are appreciated microscopically in some cases (Figure 1C). Leukocytic infiltration is not a prominent feature of the histopathology associated with *C. perfringens* type C infection [14]. Numerous Gram-positive rods that are positive for *C. perfringens* immunohistochemistry (IHC) (Figure 1D) are often noted along the mucosal surface in affected segments of intestine. The distribution of the microscopic lesions is similar to the distribution of the gross lesions and can range from diffuse to multifocal or segmental [10,13].

**Diagnosis.** A presumptive diagnosis of *C. perfringens* type C-associated disease can be made based on clinical signs in conjunction with gross and histologic lesions. This diagnosis is further supported by isolation of *C. perfringens* type C from the intestinal tract. Confirmation of the diagnosis is based on detection of CPB in intestinal content; this is typically performed using an enzyme-linked immunosorbent assay (ELISA) [9]. The presence of *C. perfringens* type C within the intestinal tract is variable and may not be isolated from all segments of intestine in affected animals. As a result, some animals with *C. perfringens* type C-associated disease may be culture negative for *C. perfringens* type C but ELISA positive for CPB [10]. It is worth noting that *C. perfringens* type C is rarely isolated from the intestinal tract of normal foals [13], and therefore the presence of this bacterium on culture warrants concern for *C. perfringens* type C-associated disease. Polymerase chain reaction (PCR) can be used for detection of toxin-producing genes in bacterial isolates. Although this technique has been also used to detect the *cpb* gene directly on feces, this has the same diagnostic significance as isolation of *C. perfringens* type C (see above).

#### 2.1.4. NetF-Associated Diseases

**Etiology.** NetF is a β-pore-forming toxin produced by certain *C. perfringens* type A strains [15]. The toxin is trypsin sensitive. It belongs to a group of necrotizing toxins, but its role in disease of equids is still under discussion. Epidemiological evidence that supports a possible role is mounting, since NetF seems to be more prevalent in the intestine of horses with enterocolitis than in healthy individuals [16,17,18].

**Epidemiology.** Net-F positive *C. perfringens* strains have been isolated from foals with necrotizing enteritis sporadically, although they have been also isolated from healthy foals [13]. Affected foals are younger than 6 days and there is no apparent breed or sex predisposition [17]. No definitive risk factors have been identified, but trypsin-inhibiting components in the colostrum have been suggested to play a role in the development of disease by *netF*-positive *C. perfringens* strains.

**Clinical signs.** Diarrhea, often times hemorrhagic, accompanied by lethargy, anorexia, and fever are the most common clinical signs of Net-F-associated enterocolitis in horses. A very sudden onset with peracute clinical course characterizes the disease in most descriptions [18].

**Pathogenesis.** NetF belongs to the leukocydin-hemolysin family of toxins [15]. Other toxins in this family have a necrotizing effect on the enteric epithelium; however, there is still not much information about the particular effects of this toxin on equine tissues.

**Gross pathology.** Fibrinous to necrotizing enteritis and enterocolitis characterize the cases of NetF-associated disease in foals. Hemorrhage is less common, although it may also occur [16,17]. On occasions, a loose pseudomembrane can be found overlying the mucosa.

**Histopathology.** In the few descriptions available, there is necrosis of the intestinal mucosa and scattered neutrophils that tend to form a band between the non-viable and viable tissue. Gram-positive bacilli can be detected attached to and within the necrotic mucosa [13,17].

**Diagnosis.** The limited information about this process complicates the diagnosis and therefore there are currently no defined diagnostic criteria available. In addition, a commercial ELISA for NetF toxin detection is not available. Isolation of NetF-positive *C. perfringens* type A from intestinal contents helps, but has no definitive diagnostic value, since this organism may be present in the intestines of many healthy horses [17,18]. In such scenarios, a presumptive diagnosis by exclusion that rules out other common causes of enterocolitis in foals is warranted [13].

#### 2.1.5. Other *C. perfringens*-Related Diseases in Horses

CPB2-toxigenic *C. perfringens* was detected more frequently in horses with enteric diseases than in control healthy animals in a study [19]. However, a description of the gross or microscopic pathology associated with this organism is not available in the study referred to [19] or elsewhere, the disease has not been reproduced experimentally, and Koch’s postulates have not been fulfilled. The role of CPB2 in enteric disease of horses remains, therefore, speculative.

### 2.2. Diseases Produced by Clostridioides difficile

#### 2.2.1. Classic *Clostridioides difficile*-Associated Disease

**Etiology**. *Clostridiodes difficile* is a spore-forming, strictly anaerobic, Gram-positive rod that causes *C. difficile*-associated disease (CDAD) via its two principal toxins, toxin A (TcdA) and toxin B (TcdB); certain strains produce also a third ADP-ribosylating binary toxin (CDT), which also contributes to disease [2]. The bacterium is ubiquitous and naturally present in the intestinal tract of some healthy horses and other animals [20,21].

**Epidemiology**. CDAD affects adult horses and foals sporadically, occasionally causing small outbreaks [22,23]. Lethality in foals can range from 0 to 42%, but in adult animals it tends to be lower [21,22]. Horses acquire *C. difficile* through ingestion of the spores or, less commonly, vegetative forms. *C. difficile* may be shed by healthy carriers, which could be as high as 29% in foals less than 14-days old [24], and from 0 to 10% in adult horses [25,26]. The cumulative prevalence in the latter age group was up to 40% over a period of 1 year in a study [27]. Spores prevail in the environment for long periods, and once ingested they germinate in the intestine. Antibiotic administration and hospitalization have been proposed as the main risk factors for CDAD development, although the role of these factors has been questioned by some authors [20,21]. Furthermore, since some cases of CDAD occur in horses that have not been exposed to hospitalization or antibiotics, other yet undetermined predisposing factors are probably important for the disease in horses.

**Pathogenesis**. Once they germinate in the intestine, vegetative forms of toxigenic strains of *C. difficile* produce TcdA and/or TcdB, which act individually or synergistically causing damage to the intestinal mucosa [28,29]. TcdA binds to multiple carbohydrate receptors in the intestinal epithelium whereas the receptors for TcdB have not been identified yet. Cytoskeletal alterations via Rho glycosylation, apoptosis, and impairment of different intracellular signaling pathways by Ras GTPase inactivation are some of the mechanisms of toxin-mediated damage at cellular level [29]. CDT may also alter the intestinal epithelium, although CDT-producing strains have been only rarely reported in horses [30].

**Clinical signs**. The clinical presentation of CDAD is similar to other causes of enterocolitis in horses and is not highly specific [13]. Diarrhea, colic, and fever are the most common clinical signs [13,20]. The clinical signs may be slightly different depending on the age: adult horses usually present with fever and abdominal pain initially [29], whereas a fulminating form of the disease with sudden onset of bloody diarrhea, depression, toxemia, and death is described in newborn foals [29].

**Figure 2:**
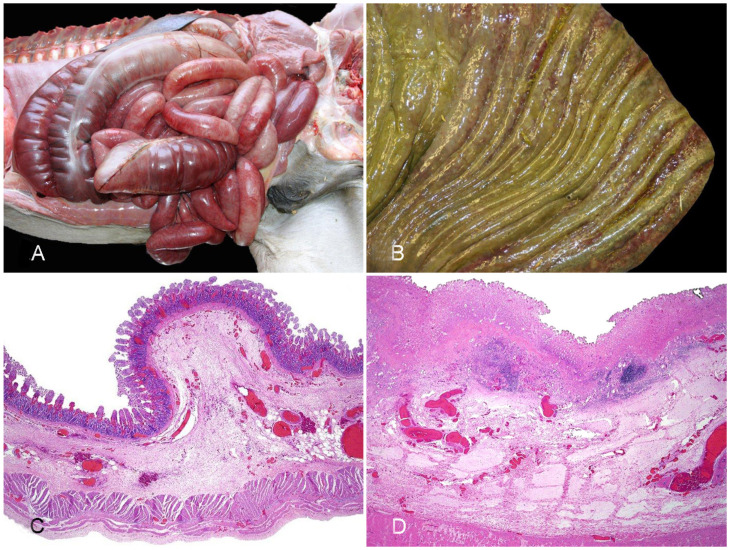
Horse with enterocolitis produced by *Clostridioides difficile*. (**A**) There is diffuse red discoloration in the serosa of most of the small intestine and colon. (**B**) The mucosa of the colon is congested and hemorrhagic, has thickened folds and it is covered by a fibrinous pseudomembrane. (**C**) The small intestine shows villus blunting, loss of superficial epithelium, congestion and hemorrhage of the mucosa and severe submucosal congestion and edema. (**D**) There is diffuse necrosis of the mucosa, which is covered by a pseudomembrane, and congestion and edema of the submucosa. Reprinted with permission from ref. [29]. Copyright 2013 Vet Pathol.

**Gross pathology**. Gross abnormalities of CDAD may be found throughout the entire intestinal tract, including small intestine, colon, and cecum. The distribution of such lesions, however, varies with the age of the affected animals [20,31]. Very young foals (i.e., less than 1 month old) tend to present intestinal lesions with a cranial distribution, with changes most often focused on the small intestine, although colon and cecum may be also affected. In contrast, colonic and cecal involvement predominate in older foals and adult horses. In both scenarios, there are soft to watery and greenish to occasionally hemorrhagic intestinal contents. There is usually a diffuse dark red discoloration in the serosa and mucosa of the affected segments, and pseudomembrane formation is frequently described (Figure 2A,B) [32]. In a recent study that examined gross and microscopic lesions in 90 cases of enterocolitis by different causes, necrosis and hemorrhage of the large intestinal mucosa was reported significantly more often in CDAD than in other diseases [14]. Thickening of the entire wall by edema with a gelatinous aspect was once considered specific of CDAD, although this may also occur with other causes of enterocolitis in horses [14]. In addition, extraintestinal lesions related to endotoxemia or septicemia such as congestion and hemorrhages in multiple organs, pulmonary edema and congestion, or hepatic necrosis may be seen [14,20].

**Histopathology**. Histologically, CDAD causes an array of changes in the intestinal mucosa similar to other causes of enterocolitis in horses [14]. There is necrosis of the surface epithelium with pseudomembrane formation, congestion and hemorrhage, variable numbers of neutrophils and mononuclear leukocytes in the lamina propria and submucosal edema (Figure 2C,D) [14,20]. Thrombosis of the proprial and submucosal vessels also occurs, and it has been reported in around 15% of the cases in a recent study [14]. The so-called volcano ulcers, characterized by a focal superficial disruption of the mucosa with release of neutrophils, fibrin, and debris to the lumen, is considered a very characteristic histological lesion of CDAD. This finding has been reported in horses [13], although to a lesser extent than in other species, probably due to the higher rate of autolysis of the mucosa in equids and/or to the advanced necrosis of the superficial epithelium, which precludes observation of ulcers [31]. Gram stain may reveal positive rods overlying or within the necrotic intestinal mucosa [13].

**Diagnosis**. A presumptive diagnosis of CDAD may be based upon clinical signs and lesions coupled with a history of antibiotic treatment and/or hospitalization [20]. Other enteric processes such as salmonellosis, other clostridial diseases or NSAIDs-mediated colitis should be included in the differential diagnosis, since their clinical, gross and microscopic findings may mimic CDAD [13,14]. Therefore, the diagnosis of CDAD needs to be confirmed via ancillary testing, preferable by detection of TcdA and/or TcdB from feces or intestinal contents using ELISA [20,33]. If bacterial isolation is requested, samples should be submitted in anaerobic transport medium; *C. difficile* grows well in cycloserine-cefoxitin-fructose-containing selective media [34], but isolation of *C. difficile* has limited diagnostic relevance, since a percentage of healthy horses may carry the bacterium [21]. However, isolation of large numbers of *C. difficile* and PCR toxinotyping of the isolates has diagnostic value [22], especially if a toxinogenic strain is detected and other causes of enterocolitis are ruled out; toxinotyping is, however, not performed as a routine test in most veterinary diagnostic laboratories.

#### 2.2.2. Other Diseases Associated with *Clostridioides difficile*

Recently, *C. difficile* has been associated with the so-called duodenitis-proximal jejunitis (DPJ) syndrome, a sporadic gastrointestinal process for which a specific cause is largely unknown [35]. DPJ was successfully reproduced by inoculation of *C. difficile* toxins via gastroscopy [36]. Affected horses may present with ileus and colic of variable severity accompanied by nasogastric reflux [35]. Grossly, experimental cases show dark red discoloration of the duodenum with variable degrees of mucosal congestion, hemorrhage, and edema. Microscopically, neutrophilic infiltrates are seen in the mucosa of the duodenum and jejunum [36]. Currently, there are no defined diagnostic criteria for *C. difficile*-associated DPJ, and the involvement of other agents such as *Salmonella* spp., other *Clostridium* spp. and mycotoxins cannot be excluded [35].

### 2.3. Enteric Disease Produced by Paeniclostridium sordelli

*Paeniclostridium sordellii* (previously known as *Clostridium sordellii*) has been recently associated with enterocolitis in horses [37]. In the study referred to, *P. sordellii* was demonstrated by culture, IHC, and/or PCR in the intestine of seven horses with enterocolitis. Grossly, these horses had necrotic, hemorrhagic, and edematous small and/or large intestine. Histopathology revealed mucosal necrosis and hemorrhage of the grossly affected parts of the small and/or large intestine. A presumptive diagnosis in these cases was established based on gross and microscopic lesions coupled with detection of *P. sordellii* in intestinal content and ruling out other known causes of enterocolitis in horses.

## 3. Histotoxic Clostridial Infections

### 3.1. Gas Gangrene

**Etiology.** Gas gangrene is caused by one or several clostridial species [38,39,40]. Although in horses the disease is most often caused by *Clostridium perfringens* type A [38,41], sporadic cases associated with other clostridial species have been described. These include: *Clostridium septicum*, *Clostridium chauvoei*, *Clostridium novyi*, *Clostridium ramosum*, *Clostridium sporogenes*, and *Clostridium fallax* [38,42,43,44,45]. Most cases of equine gas gangrene reported have been produced by a single clostridial species. However, mixed infections with two or more clostridial species have occasionally been reported [38,46,47,48]. Recently, a series of cases of gas gangrene produced by *P. sordellii* in horses was described [40]. Several of the agents responsible for gas gangrene are part of the intestinal microbiota of many animal species and can be found in the environment [49,50,51]. These clostridial species may be post-mortem invaders [52] or invade tissues during the last minutes of life [53]; therefore, caution should be taken when interpreting culture results of carcasses that are not very fresh.

**Epidemiology.** Gas gangrene of horses is a sporadic disease. The disease occurs when there is intimate contact between the clostridial species involved and animals, typically by contamination of wounds after accidents, parturition, vaccination, or other traumatic interventions [54]. The most common predisposing factor for gas gangrene in horses seems to be intramuscular injections [38,40,41,55]. Omphalitis produced by *P. sordellii* occurs in neonatal foals [54]. In other cases of gas gangrene, no association with sex or age of horses has been identified. It has been suggested that Quarter horses are more predisposed to gas gangrene than other breeds because in a study of 37 cases of equine gas gangrene, 43.2% of cases occurred in this breed [38]. This, however, has not been proved.

**Pathogenesis.** Contamination of wounds with spores or vegetative forms of clostridia is the main predisposing factor for most cases of gas gangrene. The low redox potential and metabolites of decomposing protein and acid pH in areas of trauma promote germination of spores and stimulate proliferation of vegetative forms of clostridia [56], with the consequent production of toxins. The latter are the main virulence factors of the bacteria involved and are responsible for the clinical signs and lesions of the disease. However, because cases with no evidence of wounds have rarely been reported, it has been postulated that some cases of gas gangrene in horses may have a pathogenesis similar to that of blackleg in cattle [38,48,57]. In the latter, the spores of *C. chauvoei* are ingested, absorbed into the systemic circulation and reach cardiac and skeletal muscle, amongst other tissues, where they stay dormant until injuries that do not produce skin or mucosal wounds occur. These promote an anaerobic environment where the spores germinate, proliferate and produce the toxins that are responsible for the disease [58,59]. This, however, has not been definitely proved and remains speculative. A detailed description of the mechanism of action of each histotoxic clostridial toxin is beyond the scope of this review. Briefly, most histotoxic clostridial toxins act first on endothelial cells, producing circulatory alterations, including edema and hemorrhage, leading to ischemia and local necrosis [60], which provides the ideal conditions for survival and multiplication of these microorganisms and production of more toxins [61]. The action of these toxins is enhanced by several enzymes produced by the histotoxic clostridia, including collagenases, DNAses, hyaluronidases, and neuraminidases [61,62,63]. Toxemia and bacteremia usually occur, leading to shock and death [53,61,63]. Intravascular hemolysis can occasionally be observed in cases of human gas gangrene caused by *C. perfringens* type A, due to the hemolytic nature of CPA and PFO [51]; to the best of our knowledge, this has not been observed in horses.

**Clinical signs.** Fever, tachycardia, depression, respiratory distress, muscle tremors, and anorexia are the clinical signs most frequently seen in cases of gas gangrene [64]. Soon after infection, the inoculation site and surrounding tissues are swollen, red, hot, and painful. The swelling increases as the infection progresses, aided by subcutaneous edema and emphysema; the skin becomes taut, dark red, or black. Crepitation becomes evident on palpation. Reluctance to move, lameness and eventually recumbence occur if the lesions are in the limbs [42,55]. Eventually, the affected tissues become cold [53]. Death as a consequence of toxemia and shock occurs a few hours to 3 days after the onset of clinical signs, although chronic cases lasting for up to 30 days may also occur [42,55]. Sudden death, with animals dying without clinical signs being observed, may occasionally occur [53]. In cases of omphalitis, there is swelling of the umbilicus with suppurative exudate, accompanied by signs of toxemia [54].

**Figure 3:**
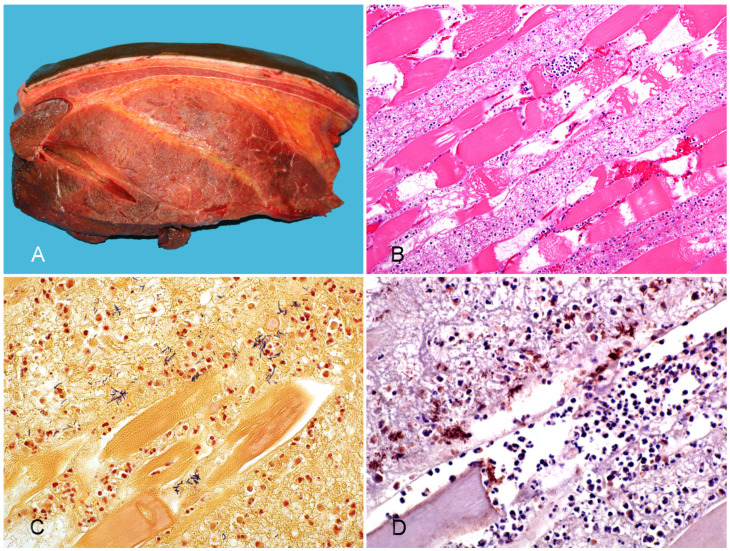
Tissues from a horse with gas gangrene produced by *Paeniclostridium sordellii*. (**A**) Severe subcutaneous and interstitial edema. (**B**) Coagulative necrosis, hemorrhage, edema, and neutrophil infiltration in skeletal muscle; HE. (**C**) Clusters of Gram-positive rods in necrotic skeletal muscle. Gram stain. (**D**) Intralesional strong positivity to *P. sordellii* in affected skeletal muscle. *P. sordellii* IHC. Reprinted with permission from ref. [40]. Copyright 2019 J Vet Diag Invest.

**Gross pathology.** Blood-stained and gelatinous subcutaneous edema and emphysema, with underlying muscles showing petechiae, echymoses, and/or multifocal to coalescing dark red, grey, or blue discoloration are the main gross changes observed (Figure 3A) [55,64]. Systemic changes include serosal and sub-endocardial hemorrhages, congested and edematous regional lymph nodes, spleen, lungs, and liver [38,40,42]. The gross findings are usually very similar, regardless of the clostridial species involved [39,53]. In a series of cases of clostridial omphalophlebitis in foals, the parietal peritoneum, subcutaneous tissue, and abdominal muscles around the internal umbilical remnant were swollen and hemorrhagic, and yellow and gelatinous edema was found in the abdominal wall, together with blood-stained and foul-smelling peritoneal fluid. Petechiae and echymoses were observed in the abdominal serosas [54].

**Histopathology.** Microscopic changes include proteinaceous edema and hemorrhage, vasculitis, and thrombosis in subcutaneous and muscular tissue [40]. Congestion, hemorrhage, emphysema, and coagulation necrosis of skin and muscle are frequently observed (Figure 3B) [65]. Leukocyte infiltration, composed mostly by neutrophils, can be minimal to severe in affected tissues. Gram-positive bacilli, single or in clusters, with sub-terminal spores are observed mostly in subcutaneous tissue and less frequently in muscle (Figure 3C) [40,65]. Circulatory disturbances, including congestion and hemorrhage of liver and lungs, and medullary hemorrhage and necrosis of thymus and regional lymph nodes, are also characteristic [40,42]. Arteritis of umbilical arteries has been observed in cases of clostridial omphalophlebitis [54]. In these cases, transmural edema, hemorrhage, and mixed inflammatory infiltrate were also observed in the urachus. Although *C. perfringens*-associated gas gangrene in humans is characterized by minimal inflammatory cell response, due to alpha toxin-induced impaired neutrophil mobility [66], this has been inconsistently described in horses and other animal species.

**Diagnosis.** A presumptive diagnosis of gas gangrene is based on clinical history, signs and gross and microscopic changes [39]. The diagnosis can be confirmed by detection of the clostridia involved in subcutaneous exudate by Gram, fluorescent antibody test, IHC (Figure 3D), anaerobic culture, and/or PCR [39,53,67]. Because several clostridial species that are normally present in the intestine may invade tissues soon before or after death, collection of samples for microbiological analysis must be performed as soon as possible after death [39,68].

### 3.2. Clostridial Hepatitis

The term clostridial hepatitis refers to those diseases produced by species of *Clostridium* in which the liver is the main affected organ [69]. In horses, Tyzzer disease (TD) is the most relevant condition that fulfills this description. Other clostridial hepatitis that can affect horses less frequently include bacillary hemoglobinuria (BH) and infectious necrotic hepatitis (INH) [69]. The following section describes the main features of these three diseases in horses.

#### 3.2.1. Tyzzer Disease

**Etiology.** Tyzzer disease (TD) is produced by *Clostridium piliforme*, an anaerobic, filamentous, and the only Gram-negative and obligate intracellular pathogenic clostridia. Virulence factors for this bacterium have not been characterized to date; however, different degrees of cytotoxicity can be induced by different isolates, suggesting that clinical forms of the disease may be induced by various strains [70].

**Epidemiology.** TD is mostly a disease of young foals (<45 d old), especially affecting immunocompromised individuals, with no sex predilection [71]. Adult horses seem to be resistant to TD, but they may be carriers of *C. piliforme* in the gastrointestinal tract, where it proliferates and passes through feces to the environment. *C. piliforme* spores are resistant to heat and common disinfectants. Coprophagy, which is common in foals < 5 week old, is indicated as a predisposing factor for TD, and it is rarely seen in animals older than that age [72]. In California, foals born between March and April were 7.2 times more likely to develop TD [73]. Similarly, 58% of the cases occurred in April and May on Kentucky horse farms [73]. In another retrospective study of cases of TD in California, most foals were autopsied during spring [71]. In addition, a higher risk for TD in horses may occur when animals are born to mares < 6 year old versus older mares, potentially suggesting that different quality of colostrum may be an additional risk factor [73].

**Pathogenesis.** The presumed pathway for the development of TD involves oral ingestion of *C. piliforme* spores from a fecal-contaminated environment. This is supported by experimental reproduction of the disease in foals following oral administration of feces from infected horses [72]. The spores would then colonize, multiply, and cause direct damage to the enterocytes of the ileum, cecum, and colon, from where they are absorbed into the blood, reaching first the liver by portal circulation, and then other organs [69,70].

**Clinical signs.** TD manifests primarily as hepatic disease and less frequently as intestinal disease [71]. These clinical signs may include jaundice, fever, diarrhea, depression, anorexia, tachycardia, tachypnea and lethargy, rapidly progressing to shock, convulsions, and death [74,75]. In most cases, however, foals are found dead with no evident clinical signs observed [71].

**Figure 4:**
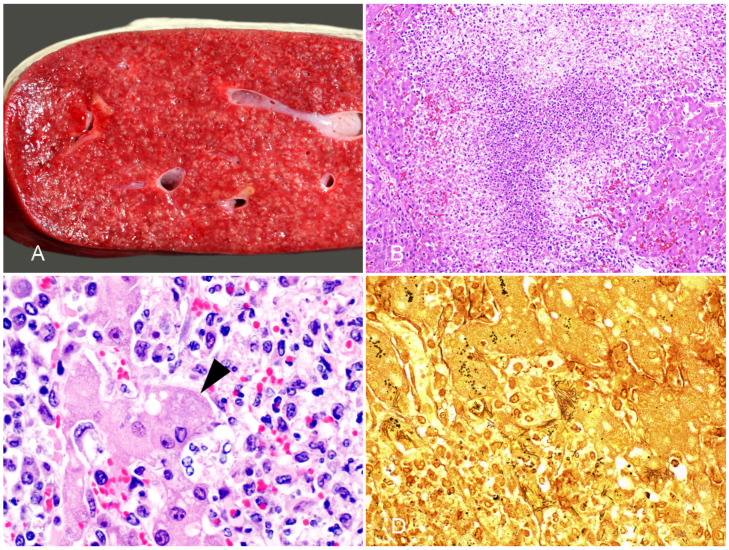
Liver from a foal with Tyzzer disease. (**A**) A cross section of the liver shows multifocal, random white spots distributed throughout the parenchyma. (**B**) Multifocal to coalescing areas of coagulative and lytic necrosis with neutrophilic infiltrate; HE. Reprinted with permission from ref. [77]. Copyright 2017 John Wiley & Sons. (**C**) Focus of necrosis showing faintly stained filamentous basophilic bacilli mostly in the cytoplasm of hepatocytes (arrowhead); HE. (**D**) Filamentous bacilli in the cytoplasm of several hepatocytes. Steiner stain.

**Gross pathology.** For most animal species affected by TD (particularly laboratory animals), a triad of lesions involving the heart, liver, and the intestinal tract is considered characteristic of the disease [69,70,75]. However, this is not always a prominent feature of TD in foals, which frequently exhibit hepatic lesions only [71]. Intestinal or cardiac lesions are only present in a small percentage of cases [71]. The liver is enlarged, and multiple, randomly distributed white pale foci are visible throughout the parenchyma (Figure 4A). Generalized icterus is a consistent feature of the disease, and widespread serosal hemorrhages are also observed in most cases. When present, gross changes in the alimentary tract are mostly present in the colon, which may be diffusely reddened, containing liquid or semi-liquid contents [71].

**Histopathology.** The microscopic features of TD are characteristic, and they consist of random foci of necrosis, demarcated and infiltrated by large numbers of viable and mostly degranulated neutrophils (Figure 4B). The cytoplasm of viable hepatocytes at the margin of these lesions contains large numbers of long, filamentous bacterial rods forming bundles or crisscross patterns, which sometimes can be seen in HE-stained sections (Figure 4C), but are best visualized with Giemsa or silver stains (Figure 4D) [69,71]. When intestinal lesions are present, they consist of necrotizing colitis, submucosal edema, and infiltration of the lamina propria with lymphocytes, plasma cells, and neutrophils. The cytoplasm of few enterocytes and/or necrotic debris may contain characteristic filamentous bacteria [69,71]. In the few cases in which the heart is involved, random foci of necrotizing myocarditis may be observed, which is characterized by myofiber degeneration and necrosis, vacuolation of myofibers, loss of striations and infiltration of neutrophils, lymphocytes and histiocytes. Intracytoplasmic filamentous bacteria are rarely detected in the heart [71].

**Diagnosis.** Epidemiological data, clinical signs, and the characteristic macroscopic and microscopic changes are highly suggestive of TD. The diagnosis must be confirmed by the detection of intracellular filamentous bacteria by means of Giemsa or silver stains in the liver [75,76] and, less frequently, in the colon and heart [71]. Since *C. piliforme* cannot be grown in conventional media, propagation in embryonated eggs is required, although this method is not commonly attempted in diagnostic laboratories [72]. PCR amplification of the 16S rRNA can also be done to confirm the etiologic diagnosis on frozen, or formalin-fixed, paraffin-embedded samples of liver [71].

#### 3.2.2. Infectious Necrotic Hepatitis

**Etiology.***Clostridium novyi* is divided into 4 types (A–D) depending on the presence of the genes that encode for alpha and/or beta toxins [69]. The traditional manifestation of IHN is produced by *C. novyi* type B, a Gram-positive, anaerobic, spore-forming rod that is commonly found in soil and the gastrointestinal content of some grazing animal species [77]. The *C. novyi* type B genotype is characterized by the presence of genes encoding for the glucosylating alpha toxin (TcnA) and the phospholipase beta toxin [63,78].

**Epidemiology.** Since few reports of *C. novyi* type B-mediated diseases in horses are available in the literature, epidemiologic and clinical information is scant [69,79]. Based on this, there is no apparent sex, age, or breed predisposition for developing INH [79]. Farms with a history of INH frequently have higher levels of *C. novyi* type B spores in the soil compared with farms with no history, and spores from dead animals contribute to contaminating pastures where animals graze [80].

**Pathogenesis.***C. novyi* spores are extremely resistant to adverse environmental stresses, and they can persist in soil for a long time [81]. The current but unproved dogma for *C. novyi*-mediated hepatitis in horses involves the ingestion of spores from soil, absorption from the intestine and latency in Kupffer cells of the liver [79]. The requirement for those latent spores in the liver to germinate is the presence of an anaerobic microenvironment, which, in ruminants, is frequently associated with necrosis produced by the migration of immature stages of liver flukes throughout the hepatic parenchyma [69]. Potentially, any cause of liver damage could be involved in this initial step of the pathogenesis, and it may include liver biopsies, abscesses, hepatic lipidosis, and toxic agents [69]. In addition, anthelmintic treatment has been suggested as a potential trigger for INH in horses, presumably by causing some degree of hepatic necrosis associated with lysis of migrating parasites [82,83]. Once anaerobiosis is established, spore germination and bacterial multiplication follows. During this stage, *C. novyi* type B produces and releases large quantities of TcnA, which is considered the main virulence factor for INH. TcnA has a monoglycosyl transferase activity that results in inactivation of GTP-binding proteins in endothelial cells, leading to cytoskeletal disruption and increased vascular permeability [78]. Vascular damage may also contribute to the formation of thrombi in multiple organs in cases of equine INH [79]. The hemolytic and necrotizing beta toxin is presumed to be responsible for inducing direct hepatic necrosis [76,77]. Since the amounts of beta toxin produced by *C. novyi* type B are low, the role of this toxin in INH is presumed to be minor compared with *C. novyi* type D (*C. haemolyticum*) in bacillary hemoglobinuria [69,84,85].

**Clinical signs.** Based on the limited information regarding INH in horses, the clinical course may last 12–72 h and it is usually fatal. Clinical signs vary among cases and may include depression and reluctance to move, ataxia, head tilt, hyperemic and/or icteric mucous membranes and ocular sclera, abdominal pain, and recumbency [83,86].

**Figure 5:**
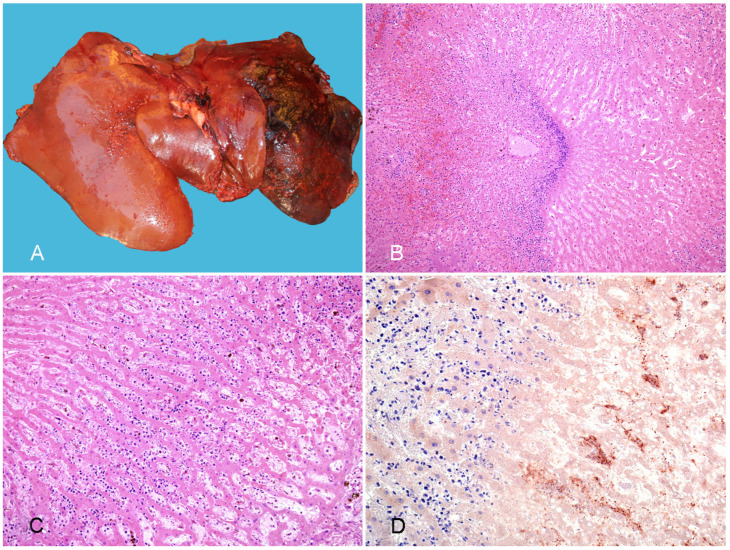
Liver from a horse with infectious necrotic hepatitis produced by *Clostridium novyi* type B. (**A**) A large focus of necrosis involves most of the left hepatic lobe. Photo reproduced from [79]. (**B**) The area of coagulative necrosis (right) is separated from an area of more viable-looking hepatic parenchyma (left) by a band of inflammatory cells; HE. Reprinted with permission from ref. [79]. Copyright 2018 J Vet Diag Invest. (**C**) Higher magnification of the band of inflammatory cells, composed mostly of neutrophils, surrounding the focus of necrosis; HE. (**D**) Strongly positive rods in the periphery of the focus of necrosis; *C. novyi* IHC.

**Gross pathology.** Horses with INH are usually in good nutritional condition. Some horses may exhibit yellow discoloration of the sclera and subcutaneous and adipose tissue. Petechiae and ecchymosis may be present in several organs. The pericardial sac and abdominal cavity may contain large amounts of serosanguineous fluid and fibrin strands [79,83]. The most consistent finding of INH in horses is, in the majority of cases, a single, well-demarcated, large focus of necrosis affecting the liver (Figure 5A), which differs from the disease in ruminants in which it is usually described as a multifocal lesion [76,77,87]. This area of necrosis may contain subcapsular and/or parenchymal gas bubbles and it may be covered by abundant fibrin over the capsule [79].

**Histopathology.** The most important finding consists of multifocal to focally extensive areas of coagulative necrosis, demarcated by a rim of mostly degranulated neutrophils, extravasated erythrocytes, cell debris, fibrin, and large numbers of Gram-positive bacilli, some of which may contain subterminal spores (Figure 5B,C). Segmental to diffuse fibrinoid necrosis of blood vessels in the liver, and fibrin thrombi in the liver and other organs may also be observed [79,87]. In some horses, the renal tubular epithelium may present cytoplasmic vacuolation, and the tubular lumen contain granular casts and proteins that stain positively with Okajima stain for hemoglobin. In some cases, diffuse congestion and expansion of the Virchow–Robin space with eosinophilic edema may be seen in the brain. In addition, lungs may be diffusely congested, with areas of hemorrhages and alveolar edema. Multiple hemorrhages may be present in the serosa of many organs [79].

**Diagnosis.** Epidemiological information including past events of the disease in the premises, clinical signs, and macroscopic and microscopic changes of INH could provide a presumptive diagnosis. Liver biopsies may be attempted to visualize histologic changes in the organ; however, such procedures and the associated liver damage may predispose and trigger the disease. Postmortem evaluation should be performed shortly after death to avoid the effects of decomposition and proliferation of clostridial species in tissues [69,83]. Given the strictly anaerobic nature of *C. novyi*, isolation is not always possible. Demonstration of *C. novyi* can be performed by IHC in association with the characteristic microscopic lesions (Figure 5D); however, currently available IHC methods are not able to distinguish among the four different types of *C. novyi*. Alternatively, PCR for methods targeting the flagellin (*fliC*) gene are available to distinguish among different histotoxic clostridial species including *C. novyi* type B, ideally on frozen samples of liver [88]. DNA amplification on formalin-fixed, paraffin-embedded (FFPE) tissues can also be attempted, but sensitivity is much lower [69].

#### 3.2.3. Bacillary Hemoglobinuria

Bacillary hemoglobinuria (BH) is a highly acute, infectious, but not contagious disease caused by *C. haemolyticum* (also known as *C. novyi* type D), which is characterized by encoding the beta toxin gene as the most relevant virulence factor, and by lacking the alpha toxin gene [69]. To our knowledge, reports of BH in horses are extremely rare. In one available report, an apparent BH case was described for this species, but the diagnosis was only presumptive, with no distinction of the type of *C. novyi* involved [82]. Given the similarities between *C. novyi* type B and *C. haemolyticum*, it is commonly accepted that both diseases follow similar epidemiologic patterns, and have analogous pathogenesis, clinical signs, and gross and microscopic changes [69]. The diagnosis of BH in horses should be based on these features, in addition to the demonstration of *C. haemolyticum* in association with the hepatic lesions. Since *C. haemolyticum* does not always grow well given its highly anaerobic nature, PCR identification is the ideal method of confirmation, by detecting the presence of the beta toxin gene, and the absence of the alpha toxin gene [69]. Alternatively, detection of the *fliC* gene for *C. haemolyticum* can be attempted [88]. Molecular studies suggest that co-infections with both *C. novyi* type B and *C. haemolyticum* are not infrequent in horses (authors’ unpublished observation).

## 4. Neurotoxic Clostridial Infections

### 4.1. Tetanus

**Etiology.** Tetanus is a neurologic disease caused by the tetanus toxin (tetanopasmin; TeNT) produced by the bacterium *Clostridium tetani* [89]. *C. tetani* is a Gram-positive, sporulating, anaerobic bacillus found within the environment. Like most other clostridia, this bacterium has been shown to germinate under low oxygen tension [90].

**Epidemiology.** While uncommon, neurologic disease as a result of TeNT intoxication is often severe with high mortality in affected animals [91,92]. Susceptibility to TeNT varies between animal species and unvaccinated horses are particularly susceptible to this neurotoxin [89].

**Pathogenesis.** Tetanus typically occurs when deep wounds are contaminated by *C. tetani* spores [89], including umbilical cord contamination in foals, puncture wounds in distal limbs, sole abscesses, rope burns, wire cuts, and tail docking [93]. In this anaerobic environment, the spores germinate and produce a number of toxins including TeNT and tetanolysin, a hemolysin thought to promote tissue colonization by causing necrosis. The spores can persist within the contaminated tissue for several weeks until conditions become favorable for germination [90]. TeNT is transported retrogradely through motor and later sensory nerves to the spinal cord, where it blocks release of neurotransmitters from the inhibitory interneurons [90,94]. Synaptobrevin, a membrane protein necessary for the release of neurotransmitters, is cleaved by TeNT thereby inhibiting the release of glycine and gamma-aminobutyric acid (GABA).

**Figure 6:**
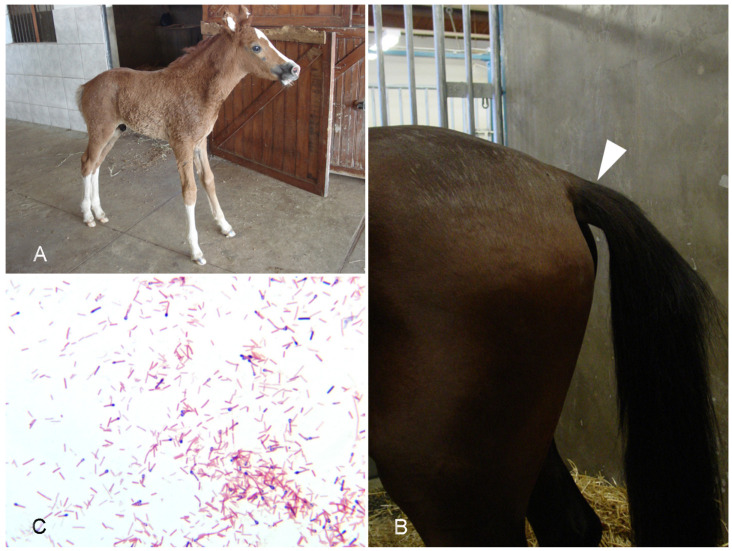
Tetanus. (**A**) Foal showing muscle stiffness resulting in a “rocking horse” stance and lock-jaw (photo courtesy of Francisco Lobato). (**B**) Adult horse; the tail is held out (arrowhead; hoto courtesy of Nicola Pusterla). (**C**) Smear of *Clostridium tetani* culture showing Gram-positive rods with terminal spores giving them the typical drumstick appearance; notice that the Gram positivity has been lost in some of these rods. Gram stain.

**Clinical signs.** Tetanus is a neurologic disorder characterized by spastic paralysis [89,95]. In horses, the paralysis often initially affects the head, then respiratory muscles and finally the limbs, leading to a typical “rocking horse” stance (Figure 6A). Prolapse of the third eyelid and held out tail (Figure 6B) are common in the early stages of tetanus in horses. Lateral recumbency, extensor rigidity, inability to ambulate, opisthotonos, muscle spasms, and trismus may be appreciated in more severely affected animals [96]. While some horses may recover, death can occur in as little as one to two days following onset of clinical signs. Several different forms of tetanus are recognized including generalized and localized tetanus [89,95].

**Gross pathology.** Postmortem findings in animals affected by tetanus are often non-specific [92]. Lesions such as ulceration secondary to prolonged recumbency may be appreciated. Examination of the carcass for a deep penetrating wound(s) is warranted in cases with a history of neurologic disease/spastic paralysis. These lesions may be difficult to find (authors’ unpublished observation).

**Histopathology.** There are no characteristic microscopic lesions associated with tetanus.

**Diagnosis.** A presumptive diagnosis of tetanus can be made based on history and clinical signs [89,96]. The absence of significant gross or microscopic post-mortem lesions supports the diagnosis. Isolation of the bacterium or observation of the typical Gram-positive bacilli with terminal spores in wounds (Figure 6C) is also supportive, but not diagnostic, as wounds can be contaminated by contact with soil. Detection of TeNT in lysates of wounds or blood by mouse bioassay used to be performed but is not currently available in most veterinary diagnostic laboratories (authors’ unpublished observation).

### 4.2. Botulism

**Etiology**. Botulism is a severe, flaccid neuroparalytic disease that affects humans and many other animal species [97]. Botulism is produced by *Clostridium botulinum*, which is classified into types A to H by the production of toxins with the same names (A through H). These botulinum neurotoxins (BoNTs) act at the neuromuscular junction, blocking the release of acetylcholine and leading to flaccid paralysis. Types B, C and D and their mosaic variants are the types most frequently involved in equine botulism worldwide, although cases caused by BoNT type A and B have also been reported [97,98,99].

**Epidemiology**. Although *C. botulinum* is a normal inhabitant of the intestinal tract of many healthy animals, this organism is rarely found in the gastrointestinal content of normal horses [97]. As said above, ingestion of pre-formed BoNT is the most common form of botulism in horses. A common scenario has been seen when forage containing BoNT or improperly preserved forage is administered to horses [100]; this has led to devastating outbreaks of the disease [99]. Some serotypes are more prevalent in certain geographic regions. For instance, in the United States, botulism type A occurs in the western states, but type B is endemic in mid-Atlantic states and Kentucky [97,100,101,102].

**Pathogenesis**. Equine botulism occurs via three possible mechanisms, the most common of which, as stated above, is ingestion of preformed toxin present in dead animals, decayed organic matter, feed, and water. The second most prevalent form is the toxico-infectious form, which occurs when the organism grows in the intestine and produces toxins, which are then absorbed. In horses, this is thought to occur in foals. Wound botulism is the third, but rare form of botulism, which results from infection of a wound in which the local anaerobic environment favors bacterial multiplication and toxin production. An example of this is the so-called shaker foal syndrome, which is believed to occur when ulcers or other necrotic lesions in 2- to 4-week-old foals are infected by *C. botulinum*. Other external wounds may also result in wound-related botulism in older horses [97].

**Figure 7:**
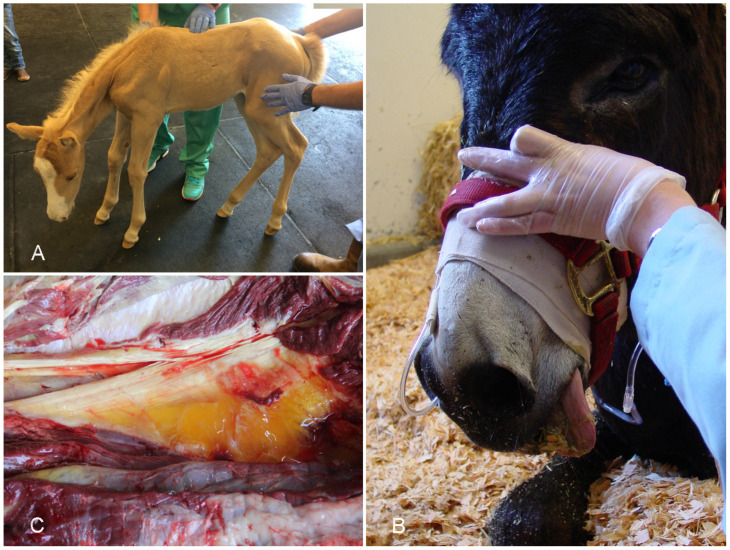
Botulism. (**A**) Foal showing muscle weakness, leading to inability to keep its head up and needing help to stand (photo courtesy of Gary Magdesian). (**B**) Adult horse with decreased muscle tone of the tongue, which is protruding outside the mouth (photo courtesy of Nicola Pusterla). (**C**) Neck of an adult horse showing gelatinous edema surrounding the nuchal ligament.

**Clinical signs.** Clinical signs of animal botulism are often strongly indicative but not specific. Botulism in most mammalian species, including horses, is characterized by progressive, symmetrical and flaccid paralysis, which often starts in the hindquarters with weaknesses (Figure 7A), muscle tremors, stumbling, and recumbence; it is usually fatal. Weakness progresses from the hindquarters to the forequarters, head, and neck. Disease ranges from peracute to chronic forms. Some cases may present as sudden death. Clinical signs appear from 18 h to 17 days after exposure. In adult horses, the first sign of botulism can be mild abdominal discomfort, generally followed by muscle weakness and dysphagia. Botulism in horses is also characterized by decreased tail, eyelid, and tongue tone (Figure 7B). This can lead to inability to withdraw the tongue into the mouth, recumbency and difficulty in rising, lifting the head, and breathing. Mydriasis is also common, notably in type C equine botulism. Horses with type A or B botulism may have more dysphagia than in type C. In the shaker foal syndrome, the initial signs are long periods of lying down and muscle tremors. Lethargy and weakness increase as the disease progresses and respiratory difficulties occur. Other signs are very similar to the ones observed in adults, but they develop more slowly [98,99,102].

**Gross pathology.** Botulism is characterized by absence of specific gross lesions. This can be used as a presumptive diagnostic criterion. Edema of the nuchal ligament has been described in horses with type A and C botulism (Figure 7C) [99]; this is thought to be a consequence of the inability of horses to keep the head up due to weakness of the neck muscles. The lesion is, however, not specific for botulism and can be seen in other unrelated conditions.

**Histopathology.** No microscopic changes are found in cases of botulism.

**Diagnosis.** Diagnosis of equine botulism is first based on clinical signs, which are indicative but not specific, coupled with laboratory confirmation. The definitive confirmation of a diagnosis of botulism requires detection and identification of the neurotoxin type. Detection of BoNTs can be performed on feed samples and/or in tissues or fluids of affected animals. The gold standard is still the mouse bioassay (MBA). Horses may be more sensitive to BoNTs than mice and this may reduce the sensitivity of the MBA. Another drawback of the MBA is the impossibility of discriminating between mosaic types and the partial cross-reactivity of type C or D antitoxin when diagnosing mosaic botulism. A variety of techniques to detect BoNTs have been developed, including ELISAs, enzymatic methods based upon BoNT cleavage of synthetic peptides followed by different detection of the specific product, or cell-based assay [103,104,105,106,107,108]. These techniques, although promising, have, however, not been validated for the diagnosis of equine botulism.

## 5. General Discussion

Most of the clostridial diseases described in other animal species may occur in horses. However, there are exceptions; an example of a clostridial disease that is highly prevalent in ruminants, but to the best of our knowledge has not been proved to occur in horses, is enterotoxemia by *C. perfringens* type D [9]. The cause of the apparent lack of occurrence of type D disease in horses is unknown; it is possible, however, that horses lack the receptor for ETX, the main virulent factor of *C. perfringens* type D. Several of the clostridial diseases that occur in other species and in horses, present in the latter with specific characteristics, have been described in this review. An example of these is gas gangrene, which in horses is most frequently produced by *C. perfringens* type A [38], but in ruminants is most commonly associated with *C. septicum* [39]. The differences of presentation between species are not fully understood, but it is possible that cell receptors and/or management practices are, at least in part, responsible for them. Based on these differences, different approaches to diagnosis, control and treatment should be considered when dealing with clostridial diseases of horses.

Over the past few years, significant advances have been made in our understanding of the pathogenesis of clostridial diseases of horses. Amongst these, discoveries of new toxins, such as NetF, have opened the doors to investigate possible new pathogenic pathways for enterocolitis. Although it is generally accepted that most strains of *C. perfringens* type A are not significant enteric pathogens of horses, we know now that NetF-positive strains may be responsible for necrotizing enteritis in foals. The only evidence in this regard, however, is epidemiological and more research is required to establish a definitive role of NetF in enteric diseases of horses. In particular, conventional and molecular Koch’s postulates need to be fulfilled before ascribing this toxin a role in the pathogenesis of necrotic enteritis of foals.

In the diagnostic field, several PCR and ELISA tests have been developed and adopted by diagnostic laboratories for detection of different clostridia and their toxin. Detection of toxins is particularly critical for the diagnosis of enteric diseases because most of the clostridia responsible for these conditions may be found in the intestinal content of normal animals, which renders mere isolation of the microorganisms of little or no diagnostic significance. The same applies to tetanus and botulism because both *C. tetani* and *C. botulinum* may be found in tissues and intestinal content of a number of clinically healthy horses. Although several ELISAs and other in vitro techniques have been developed for detection of *C. botulinum* toxins, their sensitivity and specificity is not ideal, and the MBA is still the gold standard for detection of botulinum toxins. Intense research is ongoing in several laboratories around the world to develop better techniques for the diagnosis of botulism. Currently, no commercial tests are available for the detection of *C. tetani* toxins, which makes the diagnosis of this condition challenging.

Probably, the equine clostridial diseases for which the diagnosis is most straight forward are those in the histotoxic group. For these diseases, observation of gross and microscopic lesions, coupled with detection of the offending clostridia by any of the several methods currently available (culture, PCR, FAT, IHC), is considered diagnostic.

Prevention of clostridial diseases in horses is another topic that requires additional research. While there is a barrage of vaccines to prevent several clostridial diseases in ruminants, pigs, and poultry, little is available for horses and prevention of most clostridial disease relies mostly on management practices, which are not always effective to prevent these diseases.

In summary, significant research is required to improve our understanding of the pathogenesis and diagnosis of enteric diseases of horses.

## Figures and Tables

**Table 1:** Toxinotypes of *Clostridium perfringens* (adapted from [4]).

Type	Toxin Produced					
	CPA	CPB	ETX	ITX	CPE	NetB
A	+	−	−	−	−	−
B	+	+	+	−	−	−
C	+	+	−	−	+/−	−
D	+	−	+	−	+/−	−
E	+	−	−	+	+/−	−
F	+	−	−	−	+	−
G	+	−	−	−	−	+

CPA: Alpha toxin; CPB: Beta toxin; ETX: Epsilon toxin; ITX: Iota toxin; CPE: Enterotoxin; NetB: Necrotic enteritis like B toxin.

## Data Availability

Not applicable.
